# Governing antimicrobial resistance through One Health: a comparative analysis of public health governance in China and the United Kingdom

**DOI:** 10.3389/fpubh.2026.1883787

**Published:** 2026-07-16

**Authors:** Zihan Cai, Hua He, Hua Wu, Jinxin Zhao, Guanghui Zhang, Jiabao Xing

**Affiliations:** 1 School of Government, University of Birmingham, Birmingham, United Kingdom; 2 Department of Pharmacology and Toxicology, College of Veterinary Medicine, Henan Agricultural University, Zhengzhou, China; 3 Department of Microbiology, Monash Biomedicine Discovery Institute, Monash University, Clayton, VIC, Australia; 4 School of Public Health, Key Laboratory of Public Health Safety, Ministry of Education, Fudan University, Shanghai, China

**Keywords:** antimicrobial resistance, China, national action plans, One Health, public health administration, United Kingdom

## Abstract

Antimicrobial resistance (AMR) has emerged as one of the most pressing threats to global health and a major challenge for contemporary public health governance. As the drivers of AMR span human, animal, and environmental systems, the One Health framework has become central to the design of effective containment strategies. This study compares AMR governance in China and the United Kingdom (UK), focusing on institutional organization, regulatory frameworks, surveillance systems, and mechanisms for cross-sectoral coordination. The analysis highlights two contrasting governance models within the broader One Health paradigm. China relies on strong state-led coordination and hierarchical administrative mobilization, whereas the UK emphasizes specialized institutional collaboration supported by integrated surveillance infrastructures. Despite these structural differences, both countries continue to face major challenges in environmental surveillance, intersectoral data integration, and long-term coordination across sectors. Future progress in AMR control will depend not only on strengthening multisectoral governance capacity, but also on improving the integration of environmental dimensions into existing surveillance and response systems. The comparative experiences of China and the UK offer broader insights into the development of adaptive and globally coordinated One Health strategies for AMR governance.

## Introduction

Antimicrobial resistance (AMR) has become one of the most urgent challenges for global health and public health governance. In 2019, bacterial AMR directly caused an estimated 1.27 million deaths and was associated with approximately 4.95 million deaths worldwide (1). AMR reduces the effectiveness of antimicrobials, leads to treatment failure, prolongs illness, and increases the risk of severe complications. Beyond individual treatment failure, AMR also places pressure on health systems by increasing the need for diagnostic testing, infection prevention and control measures, patient isolation, second-line or last-resort antibiotics, and longer hospital stays. Outside the health sector, AMR can also affect food production, soil and water resource environment, animal health, trade, household income, and workforce productivity. For these reasons, AMR containment has become a central priority in global public health governance (2, 3).

At the global level, AMR governance has increasingly been organized through national action plans (NAPs) aligned with the WHO Global Action Plan on AMR adopted by the World Health Assembly in 2015. The Global Action Plan called on countries to strengthen awareness, surveillance, antimicrobial stewardship, infection prevention and control, and investment in sustainable AMR responses. However, the development of NAPs is not only a matter of producing policy documents. Effective implementation depends on governance structures that can define responsibilities, coordinate human, animal, and environmental sectors, integrate surveillance data, regulate antimicrobial use, and evaluate progress over time.

China and the United Kingdom (UK) provide two contrasting examples of how AMR NAPs can be translated into One Health governance. China issued the National Action Plan to Curb Bacterial Resistance (2016–2020), followed by the National Action Plan for Containing Animal-Derived Bacterial Resistance (2017–2020) and the National Action Plan for Containing Antimicrobial Resistance (2022–2025). These plans have progressively expanded AMR governance from clinical antimicrobial stewardship to a broader cross-sectoral framework involving health, agriculture, veterinary medicine, drug regulation, and environmental management. The UK began with its national AMR strategy in 2013 and subsequently developed successive five-year national action plans, including the current 2024–2029 plan, supported by specialized agencies, surveillance platforms, stewardship targets, and One Health reporting mechanisms.

This review therefore compares AMR governance in China and the UK with a specific focus on NAP development, institutional organization, regulatory frameworks, surveillance systems, and mechanisms for cross-sectoral coordination. Rather than treating AMR as one issue within a broad infectious disease agenda, we examine how the two countries implement AMR NAPs through different One Health governance models. China represents a state-led and hierarchically coordinated model that can mobilize national administrative capacity, whereas the UK represents a specialized institutional model supported by technical agencies, data systems, and professional stewardship networks. Comparing these two models can generate practical lessons for strengthening AMR containment, improving intersectoral data sharing, and building more adaptive One Health governance systems.

## The development and conceptual connotations of One Health

### The origins and development of the One Health concept

**Figure 1:**
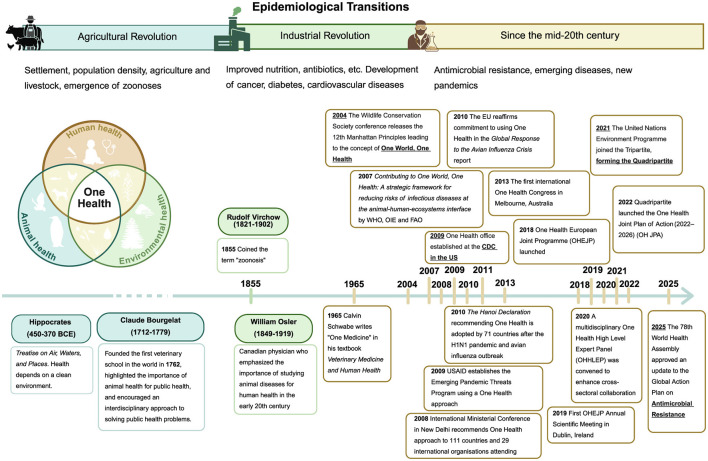
Historical evolution of the One Health concept and major milestones in its development (10). The upper panel summarizes the broader epidemiological and socio-industrial transitions associated with different historical periods, including the agricultural revolution, industrial revolution, and post-mid-twentieth-century globalization era, highlighting their implications for zoonotic emergence, chronic diseases, antimicrobial resistance, and emerging pandemics. The lower panel presents a chronological timeline of major conceptual, institutional, and policy milestones that shaped the development of the One Health framework, including foundational contributions from early scholars, the establishment of international One Health initiatives, and the progressive integration of One Health into global public health governance and antimicrobial resistance strategies.

The origins of the One Health concept can be traced back to ancient Greece, where Hippocrates proposed that health is shaped by multiple environmental factors, including water, air, and soil (Figure 1) (4). In the nineteenth century, Rudolf Virchow further advanced this idea by introducing the concept of zoonosis and emphasizing the close interconnection between human and animal medicine (5). By the twentieth century, these ideas had evolved into the concept of One Medicine (Figure 1). Looking back at the history of emerging and re-emerging infectious diseases, it is evident that the vast majority are closely linked to interactions at the human–animal–environment interface, highlighting the enduring relevance of the One Health perspective (Figure 1). Against this background, the outbreak of SARS at the beginning of the 21st century drew renewed global attention to the importance of integrated public health approaches (3, 6, 7). In 2004, the Wildlife Conservation Society convened an international conference under the theme One World, One Health, marking an important step in the development of the concept by explicitly incorporating ecosystem health into the broader health agenda (8, 9). In 2009, the U.S. Centers for Disease Control and Prevention (CDC) established the One Health Office (Figure 1), with a mission that included preventing infectious disease outbreaks, reducing infections caused by antimicrobial-resistant organisms, and improving food safety to promote the health and wellbeing of both humans and animals (10). Over the following two decades, One Health gained increasing international recognition and became progressively institutionalized within global health governance. A major milestone was reached in 2021 with the establishment of the One Health Quadripartite (OHQ), comprising the World Health Organization (WHO), the World Organization for Animal Health (WOAH), the Food and Agriculture Organization of the United Nations (FAO), and the United Nations Environment Programme (UNEP). The Quadripartite also convened the One Health High-Level Expert Panel (OHHLEP). In 2022, it released the One Health Joint Plan of Action (2022–2026) (Figure 1), which explicitly identified One Health as a central strategy for addressing major global health challenges and set out concrete action priorities in zoonotic diseases (10–13), AMR, food safety, and environmental health (3, 14).

### The core connotations of One Health

**Figure 2:**
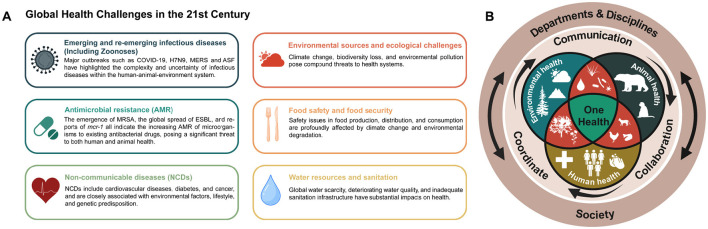
Core principles and major workstreams of the One Health approach. **(A)** Global health challenges in the 21st century; **(B)** The primary One Health collaboration framework.

In 2007, the One Health Initiative Task Force of the American Veterinary Medical Association (AVMA) refined the definition of One Health, describing it as a multidisciplinary and multisectoral collaborative approach that recognizes the interconnection and interdependence of humans, animals, plants, and their shared environment (15–17). The One Health Office of the U.S. CDC similarly emphasizes the practical application of this approach, defining its mission as preventing outbreaks of emerging and re-emerging infectious diseases, reducing infections caused by antimicrobial-resistant organisms, and improving food safety in order to protect the health of both humans and animals (14, 15). In 2017, the WHO further framed One Health as an integrated approach spanning programme design, implementation, policy, legislation, and research, with the aim of improving public health through communication and collaboration across sectors. In this formulation, priority areas include infectious disease prevention and control, AMR containment, and food safety (18). A more comprehensive and authoritative formulation was released in December 2021 by the OHHLEP under the OHQ, which defined One Health as an integrated and unifying approach that seeks to sustainably balance and optimize the health of people, animals, and ecosystems (Figure 2B). This framework emphasizes collaboration across sectors, disciplines, and communities at all levels of society to address health threats arising at the human–animal–ecosystem interface (19). It also identifies six major domains of action, including the prevention and control of infectious diseases in humans AMR containment, food safety, and environmental health, particularly emerging and re-emerging infectious diseases (Figure 2A) (3).

### Structural challenges posed by One Health to public health governance

The One Health approach poses profound structural challenges to traditional public health systems, which have historically been centered on the prevention and control of human disease. It requires a shift from reactive response to proactive early warning, as well as the development of an integrated risk-governance framework spanning the human–animal–environment interface (20). First, in many countries, existing legal and institutional frameworks remain ill-equipped to respond to cross-sectoral health threats. These limitations reduce the effectiveness of joint prevention and control efforts for complex challenges such as AMR and food safety. There is therefore an urgent need for more comprehensive legal frameworks that reflect the principles of One Health, clearly delineate the responsibilities of sectors such as health, agriculture, and ecological environment, and provide an institutional basis for coordinated action (20). Second, surveillance systems for infectious diseases, zoonotic diseases, and AMR are often organized separately within existing public health structures. At the same time, antibiotic stewardship frequently lacks effective cross-sectoral coordination, while mechanisms for interdepartmental data sharing and information exchange remain weak. Communication across sectors is often inefficient, further constraining timely risk identification and joint intervention (21–23). Together, these weaknesses hinder the translation of One Health from a guiding concept into operational practice (24). Against this background, building a modern public health governance system that is aligned with the principles of One Health has become a central priority in global health reform. This requires stronger coordination across sectors, domains, and disciplines, together with institutional arrangements capable of supporting integrated prevention, surveillance, and response.

## Public health governance systems in China and the UK

### Overview of the development of One Health governance in public health

**Figure 3:**
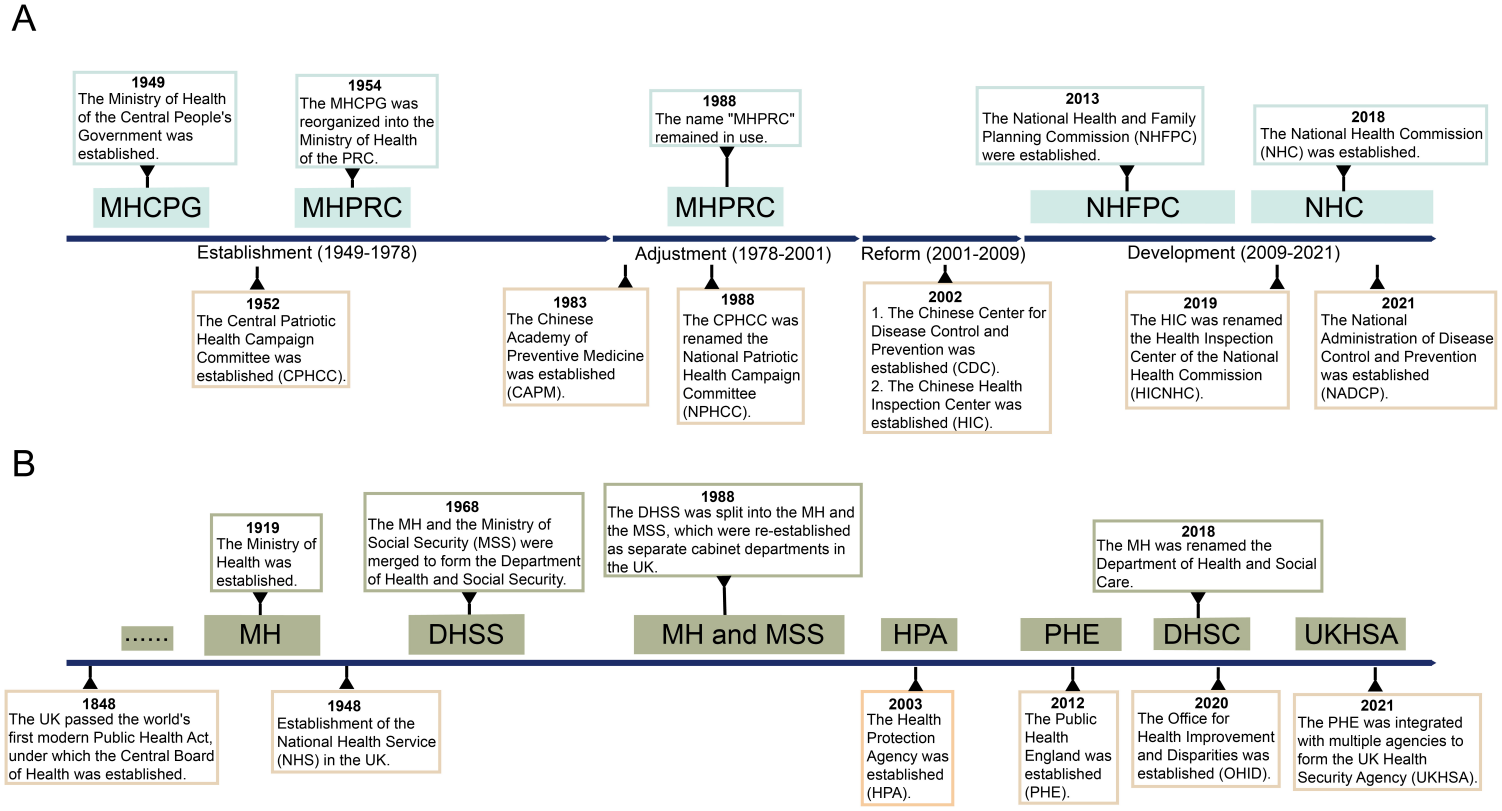
Development and Evolution of Public Health Governance Structures in China and UK. **(A)** Summarizes the historical evolution of China's public health governance system from 1949 to 2021, highlighting major institutional transitions, administrative restructuring, and the gradual incorporation of One Health-related governance concepts into national public health strategies. The development process is broadly divided into four stages: establishment (1949–1978), adjustment (1978–2001), reform (2001–2009), and development (2009–2021). Key milestones include the establishment of the Chinese Center for Disease Control and Prevention (China CDC) in 2002, the creation of the National Health Commission (NHC) in 2018, and the establishment of the National Administration of Disease Control and Prevention (NADCP) in 2021. The timeline also highlights the increasing institutionalization of disease surveillance, public health coordination, and health-centered governance in response to major infectious disease challenges and broader health system reforms. **(B)** Illustrates the historical development of public health governance structures in the UK from the 19th century to 2021. The timeline outlines the progressive evolution of national public health institutions, beginning with the establishment of the Public Health Act in 1848 and the National Health Service (NHS) in 1948, followed by subsequent institutional reorganizations aimed at strengthening infectious disease control, health protection, and intersectoral coordination. Key milestones include the establishment of the Health Protection Agency (HPA) in 2003, Public Health England (PHE) in 2012, and the UK Health Security Agency (UKHSA) in 2021. Together, these developments reflect the UK's long-standing emphasis on coordinated health protection systems and increasingly integrated approaches to infectious disease preparedness, antimicrobial resistance, and health security governance consistent with the principles of One Health.

The development of public health governance in China can broadly be divided into four stages: the establishment period (1949–1978), the adjustment period (1978–2001), the reform period (2001–2009), and the development period (2009–2021) (Figure 3A) (25). In its early phases, China's public health system was primarily oriented toward human disease prevention and mass prevention and control. With the deepening of reform and opening up, however, the system became increasingly institutionalized and legalized (26). Major outbreaks such as SARS and avian influenza prompted growing recognition of the need for more integrated approaches to health governance. In 2002, China established the Chinese Center for Disease Control and Prevention (China CDC) (Figure 3A), strengthening the institutional basis for national disease control. In the academic sphere, the One Health Foundation was established at Fudan University in 2013, and the first international One Health forum in China was held at Sun Yat-sen University in 2014, marking the entry of One Health into China's formal academic and policy discourse (27, 28). Between 2009 and 2019, China undertook major health system reforms and introduced a range of laws and regulations to strengthen public health governance (29). Institutional reform in 2018 led to the establishment of the National Health Commission (NHC) (Figure 3A), reinforcing a broader transition from treatment-centered administration toward health-centered governance (30). This shift was further consolidated in 2019 through the promulgation of the Basic Healthcare and Health Promotion Law, which formally established health-centered governance as a national legal principle. At the same time, One Health-related institutional capacity continued to expand. The China One Health Alliance was founded in 2021 (31), and multiple One Health research centers were subsequently established across universities, research institutions, and the China CDC (32). Taken together, these developments suggest that China has gradually moved toward a governance model characterized by stronger cross-sectoral coordination in infectious disease control, AMR surveillance, and health-related technological development (33).

The UK followed a markedly different but equally influential trajectory. It was among the earliest countries to establish formal public health institutions, beginning in the 1830s (34), and enacted the Public Health Act in 1848, widely regarded as the world's first modern public health law (Figure 3B) (35). Subsequent revisions to the Act in response to cholera, measles, scarlet fever, and typhoid outbreaks during the 19th century laid the foundations of the modern British public health system. The creation of the National Health Service (NHS) in 1948 represented a major institutional innovation and further consolidated the UK's public health infrastructure (Figure 3B). In 2003, the Health Protection Agency (HPA) was established to strengthen protection against infectious disease threats (36). Over time, the UK also developed strong traditions of interdepartmental coordination, particularly between the Department for Environment, Food & Rural Affairs (Defra) and public health agencies in areas such as animal disease surveillance and zoonotic disease control, alongside mechanisms such as Rapid Response Teams (RRTs) (37). This trajectory culminated in the establishment of the UK Health Security Agency (UKHSA) in 2021 (Figure 3B), which further institutionalized the UK's emphasis on specialized coordination, surveillance, and health security. With responsibilities spanning infectious disease threats, AMR, environmental health, and broader health protection functions, UKHSA embodies a governance model that aligns closely with the interdisciplinary and cross-sectoral logic of One Health. Beyond its domestic significance, the UK's public health governance model has also exerted considerable international influence. Many countries and regions, including parts of Europe, the Americas, and Southeast Asia, have borrowed from, adapted, or further developed elements of the British model (38–40). This diffusion highlights the UK's enduring role in shaping the broader trajectory of modern public health governance.

### AMR prevention and control systems in the two countries

**Figure 4:**
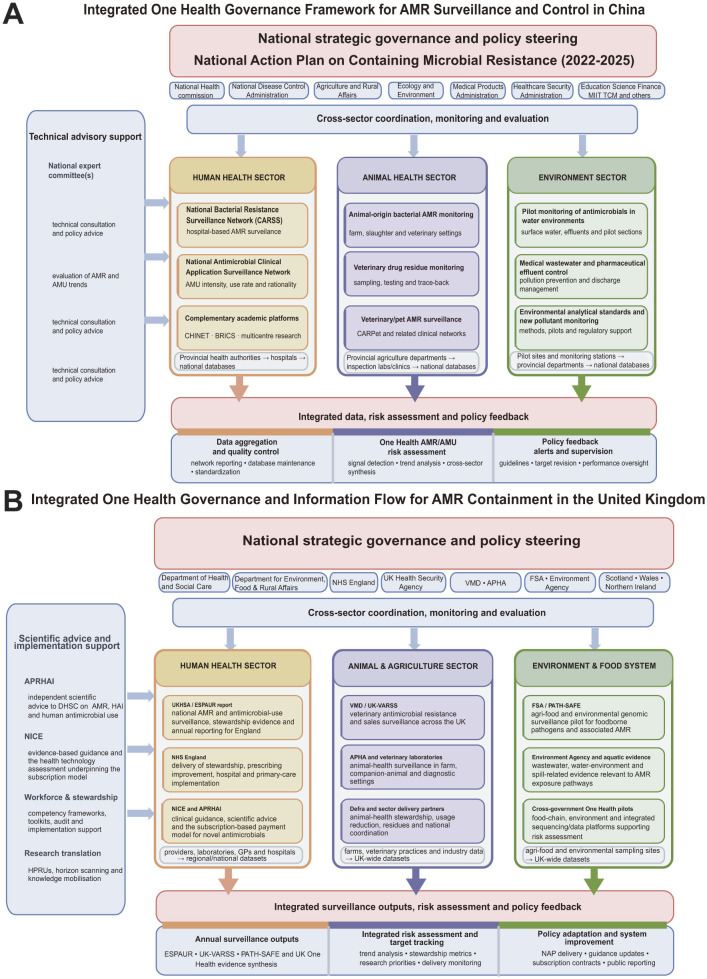
Integrated One Health governance and framework spanning Human-Environment-Animal systems for AMR surveillance and containment in China and UK. **(A)** Conceptual framework of the integrated One Health governance structure for AMR surveillance and control in China. The framework is organized around national strategic governance and policy steering under the National Action Plan on Containing Microbial Resistance (2022–2025), coordinated through cross-sectoral governmental collaboration involving health, agriculture, environmental, pharmaceutical, and regulatory authorities. The human health, animal health, and environmental sectors operate through distinct but interconnected surveillance and monitoring systems, including hospital-based AMR surveillance, veterinary antimicrobial monitoring, and environmental pollutant surveillance. Technical advisory committees and expert consultation mechanisms provide scientific support for policy development, AMR/AMU evaluation, and implementation. Data generated across sectors are integrated through centralized aggregation, risk assessment, and policy feedback mechanisms to support national surveillance, early warning, and regulatory responses. **(B)** Integrated One Health governance and information flow for AMR containment in the UK. The UK framework emphasizes specialized institutional coordination and evidence-based implementation across human health, animal and agricultural systems, and environmental and food surveillance sectors. National governance is jointly coordinated by health, agricultural, environmental, and food safety agencies through cross-sector monitoring and evaluation mechanisms. Sector-specific surveillance platforms, including ESPAUR, UK-VARSS, PATH-SAFE, and environmental monitoring programmes, contribute to integrated surveillance outputs and cross-sectoral risk assessment. Scientific advisory bodies, stewardship programmes, and implementation networks support evidence synthesis, prescribing optimization, veterinary antimicrobial stewardship, and policy adaptation. Integrated surveillance outputs are translated into policy feedback, stewardship targets, and iterative updates to the UK NAP and related AMR governance strategies.

China was among the earlier WHO Member States to establish systems for AMR surveillance and antimicrobial stewardship. In 2001, it issued the Guidelines for the Clinical Use of Antimicrobial Agents, which provided an initial institutional basis for standardizing antimicrobial use in clinical settings (Figure 4). Since then, China has developed an AMR prevention and control system characterized by strong administrative leadership, vertical coordination, and a hierarchical governance structure. At the national level, the NHC is responsible for overseeing the clinical use of antimicrobial agents in medical institutions, while the National Disease Control and Prevention Administration (NDCPA) and the China CDC are responsible for the development of AMR surveillance systems and related data analysis. Within the agricultural sector, relevant departments under the Ministry of Agriculture and Rural Affairs (MARA) oversee veterinary AMR surveillance and control. The Ministry of Ecology and Environment (MEE) is responsible for monitoring antimicrobial contamination and environmental pathways of AMR transmission, while the National Forestry and Grassland Administration (NFGA) undertakes surveillance and risk assessment of wildlife-associated AMR. This governance structure is supported by an expanding technical surveillance network. The China Antimicrobial Resistance Surveillance System (CARSS), for example, covered more than 2,217 core member institutions across all 31 provincial-level administrative regions by the end of 2024 (41, 42). It also includes the China Antimicrobial Surveillance Network (CHINET), led by Fudan University, which currently covers 78 sentinel hospitals nationwide. Additional platforms include the National Database for Animal-Origin Bacterial Antimicrobial Resistance Surveillance and the China Antimicrobial Resistance Surveillance Network for Pets (CARPet). Taken together, these mechanisms have enabled health, agriculture, and ecological and environmental authorities to establish preliminary systems for data sharing, interagency coordination, and collaborative governance in antimicrobial use management, residue monitoring, and AMR surveillance under the One Health framework. The UK, by contrast, has developed an AMR prevention and control system characterized by a more specialized and collaborative governance model. Since 2013, it has issued a national AMR strategy and repeatedly updated its corresponding five-year action plans (43). The main institutions involved include the Department of Health and Social Care (DHSC), the UKHSA, the Defra, and Defra's executive agency, the Veterinary Medicines Directorate (VMD).

Within this system, the DHSC is responsible for national AMR strategy and antimicrobial stewardship in the human health sector, while UKHSA serves as the main technical agency for AMR surveillance, analysis, and risk assessment in humans. Defra oversees animal- and environment-related AMR surveillance and early warning (44), whereas the NHS is responsible for implementing antimicrobial stewardship in clinical practice. In the veterinary sector, the VMD regulates veterinary antimicrobial use and leads AMR surveillance activities (Figure 4B). The UK system is further supported by a set of highly developed surveillance and reporting platforms. The English Surveillance Programme for Antimicrobial Utilization and Resistance (ESPAUR) is the central clinical AMR surveillance and feedback platform within the UK public health system. It connects government agencies, professional organizations, and laboratory networks across the country and includes more than 30 member organizations. Another key component is the UK Veterinary Antibiotic Resistance and Sales Surveillance (UK-VARSS) system, which monitors the sales, use, and resistance patterns of veterinary antimicrobials. In addition, the UK One Health Report, jointly issued by the VMD and UKHSA, integrates AMR data from humans, animals, food, and the environment within a unified analytical framework. Collectively, these arrangements have produced a One Health-oriented AMR governance model that relies on specialized institutions, strong technical autonomy, standardized procedures, and cross-sectoral information sharing.

## AMR prevention and control practices and outcomes in the two countries

**Figure 5:**
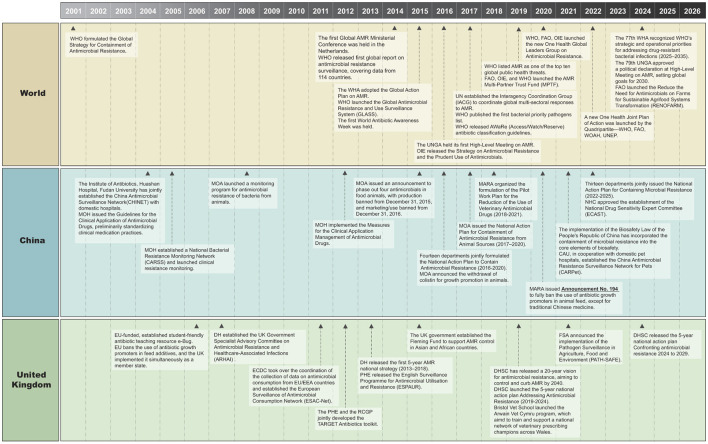
Timeline of major action plans and policy measures for AMR containment at the global level and in China and the UK, 2001–2026. The timeline summarizes key milestones in AMR governance across three levels: global initiatives led by international organizations, national policy responses in China, and national policy and surveillance developments in the UK. The global timeline highlights major developments including the WHO Global Strategy for Containment of Antimicrobial Resistance, the Global Action Plan on AMR, the establishment of the Quadripartite collaboration, and subsequent high-level political declarations and strategic action plans. The China timeline presents major national actions, including the establishment and expansion of AMR surveillance networks, clinical and veterinary antimicrobial stewardship policies, restrictions on antimicrobial use in agriculture and aquaculture, and successive National Action Plans on AMR. The UK timeline summarizes major institutional, surveillance, and stewardship milestones, including national AMR strategies, ESPAUR and UK-VARSS reporting, TARGET Antibiotics, PATH-SAFE, and the updated national action plan for 2024–2029. Together, the figure illustrates the progressive institutionalization of AMR governance and the increasing alignment of national AMR responses with the One Health agenda.

The WHO issued the Global Strategy for Containment of Antimicrobial Resistance in 2001, marking the first formal global call to address AMR (Figure 5). In 2015, the 68th World Health Assembly adopted the Global Action Plan on Antimicrobial Resistance, which emphasized the establishment of global surveillance systems and urged Member States to develop national action plans. In response, both China and the UK introduced national strategies and a range of policy measures to address the growing threat of AMR.

### AMR governance practices and outcomes in China

Following the joint issuance of the *National Action Plan to Curb Bacterial Resistance (2016–2020)* by 14 ministries and commissions in 2016, China substantially strengthened antimicrobial stewardship and AMR surveillance in both human medicine and veterinary practice. Shortly thereafter, the agricultural authorities released the *National Action Plan for Containing Animal-Derived Bacterial Resistance (2017–2020)*. In 2022, 13 departments jointly issued the *National Action Plan for Containing Antimicrobial Resistance (2022–2025)*, marking a shift from the regulation of medical institutions alone toward cross-sectoral collaborative governance spanning both human and animal health. At the same time, the scope of governance expanded from bacterial resistance to antimicrobial resistance more broadly, while environmental pollution control, pharmaceutical retail regulation, and prescription-based veterinary drug sales were incorporated into a more unified governance framework.

In terms of governance outcomes, China has made notable progress in both AMR containment and antibiotic use regulation (45). According to data from the NHC, the rate of antibacterial use in outpatient settings declined from 19.4% in 2010 to 8.1% in 2017; the antibiotic use rate among hospitalized patients fell from 59.40% in 2011 to 36.00% in 2019; and the intensity of antibacterial use among inpatients decreased from 77.6 defined daily doses (DDDs) to 45.7 DDDs. Compared with 2012, by 2018 the national surveillance network reported that, among its core member institutions, the inpatient antibiotic use rate had decreased by 7.84 percentage points, the outpatient antibiotic prescription rate had fallen by 6.2 percentage points, and the average intensity of inpatient antibiotic use had declined by 17.8%; in addition, the detection rates of 8 of the 13 major antimicrobial-resistant pathogens had decreased. A systematic study of AMR trends from 2014 to 2022 further showed that, among 383 bacterial isolates collected from 31 provincial-level regions in China, the AMR rate declined from 49.1% in 2014 to 35.8% in 2022 (46). In the area of animal-origin AMR governance, pursuant to the National Action Plan for Containing Animal-Derived Bacterial Resistance (2017–2020), the MARA issued Announcement No. 194 in 2019 (47), requiring that, from 1 January 2020, all antimicrobial growth promoters in feed additives, except traditional Chinese veterinary medicine additives, be discontinued. Commonly described as the strictest antibiotic ban, this measure represented a landmark intervention for reducing antimicrobial exposure in animals and yielded more direct results in source-oriented governance. National veterinary antimicrobial use, measured in terms of pure active ingredient weight, declined from 41,800 tons in 2017 to 32,500 tons in 2021, representing a reduction of approximately 22.2%. By 2021, China had fully phased out the use of antimicrobial growth-promoting feed additives. By the end of 2022, a total of 316 farms had participated in nationwide antimicrobial reduction pilot programmes, of which 223 met the required standards. In addition, public welfare campaigns and livestream activities promoting the rational use of veterinary antimicrobials had attracted a cumulative audience of 3.463 million views. Meanwhile, the surveillance network for animal-origin AMR continued to expand. By the end of 2018, the national clinical antimicrobial application surveillance network and the bacterial resistance surveillance network had expanded to 3,472 and 1,429 hospitals, respectively. Overall, China has developed a comprehensive AMR governance framework centered on rational antimicrobial use management, nationwide surveillance and early warning, reduction of animal-source antimicrobial use, and multisectoral coordination, and this framework has already produced measurable results.

Furthermore, although China has demonstrated strong responsiveness in areas such as surveillance and optimization of antimicrobial use, evident shortcomings remain in accountability and sanctions, regulatory sustainability, effectiveness evaluation, and environmental governance (48). Future progress in AMR containment will depend on strengthening environmental surveillance, improving the efficiency of cross-sectoral data sharing and coordination, and enhancing the targeted management and control of high-risk drug-resistant pathogens in severe infections, particularly carbapenem-resistant organisms (49). In addition, considerable regional disparities exist in China's livestock farming practices and environmental conditions. Current AMR control policies are effective in most large-scale farms, but regulatory oversight remains insufficient for some small farms in remote areas, where the continued use of banned antibiotics still occurs (50). Strengthening antimicrobial use management and AMR surveillance in small-scale farms and backyard farming systems in less developed regions will therefore be one of China's key priorities in further strengthening and effectively containing AMR in the future through measures such as economic incentives or policy-based compensation mechanisms (Table 1).

**Table 1:** Key AMR Surveillance Findings from CHINET 2024 and CARSS 2024.

Source	Dataset/denominator	AMR indicator	Key finding
CHINET 2024	458,271 clinical isolates from 74 hospitals	MRSA detection in *S. aureus*	28.4%; no vancomycin-resistant *Staphylococcus* strains reported
CHINET 2024	CHINET trend data for *K. pneumoniae*	*K. pneumoniae* resistance to imipenem/meropenem	22.6%/23.4%
CHINET 2024	CHINET antimicrobial susceptibility data	*A. baumannii* resistance to imipenem/meropenem	64.5%/64.7%
CARSS 2024	625,778 *S. aureus* isolates	MRSA detection in *S. aureus*	28.4% (177,823/625,778)
CARSS 2024	1,072,443 *K. pneumoniae* isolates	Carbapenem-resistant *K. pneumoniae*	10.0% (107,566/1,072,443)
CARSS 2024	565,812 *P. aeruginosa* isolates	Carbapenem-resistant *P. aeruginosa*	16.0% (90,491/565,812)
CARSS 2024	393,694 *A. baumannii* isolates	Carbapenem-resistant *A. baumannii*	52.2% (205,562/393,694)

CHINET reports resistance patterns from a sentinel hospital network, whereas CARSS reports national detection rates using species-specific denominators (51). CARSS carbapenem-resistant categories indicate resistance to any relevant carbapenem included in the report definition (52).

### AMR governance practices and outcomes in the UK

The UK was among the earlier countries to systematically apply the One Health approach to AMR governance. Beginning in 2013, it released a national AMR strategy, and in 2019 launched a series of five-year national action plans (Figure 5). It also developed cross-sectoral collaborative governance strategies, emphasizing the reduction of unnecessary antibiotic use in human healthcare, livestock production, and the environment. In 2017, the WHO proposed classifying antibiotics into three categories, Access, Watch, and Reserve—(AWaRe) classification. In 2019, England took the lead in completing the first England-adapted AWaRe classification, and by 2025 a unified UK-AWaRe antibiotic classification had been established across the UK (Figure 5). The current second five-year National Action Plan (2024–2029) breaks AMR work down into actionable targets, such as reducing total human antimicrobial use by 5% by 2029 relative to 2019, and ensuring that 70% of human antibiotics used come from the Access category (51). The UK has also achieved notable progress in AMR prevention and control and the rational clinical use of antibiotics. According to the 2023 One Health Report, the UK used a total of 706 tons of antimicrobials in 2019, of which 68% were for human use and 32% for animal use, representing an overall decrease of 28% compared with 2014 (51% reduction in animal use and 13% reduction in human use). The ESPAUR surveillance report showed that in 2024, antimicrobial use in NHS primary care remained below the 2019 level. The UK-VARSS reports published in 2025/2026 indicated that antimicrobial sales for food-producing animals had fallen from 36.0 mg/kg in 2014 to 15.6 mg/kg in 2024, a reduction of approximately 56.7%. During 2014–2019, the use of highest-priority critically important antimicrobials (HP-CIAs) in animals declined by 75%, from 0.38 mg/kg to 0.06 mg/kg. Particularly noteworthy is that, starting in 2023, the UK promoted an antimicrobial take-back scheme for companion animals, encouraging pet owners to return unused or expired antibiotics to professional veterinary facilities for safe disposal, so as to reduce misuse and environmental contamination and thereby help contain AMR; at the same time, requirements for medicine return were strengthened at the level of veterinary clinics (52). Although the UK is at the forefront of global antibiotic regulation and offers valuable experience for international adaptation and reference, the latest surveillance data indicate that the human burden of AMR is still increasing (53). According to figures released by the UKHSA in 2025, there were 20,484 cases of antimicrobial-resistant bloodstream infections in England in 2024, representing a 9.3% increase compared with 2023; the estimated number of 30-day all-cause deaths associated with resistant infections was 2,379. The report also showed that in 2024, 22% of antibiotics were prescribed through the private sector, suggesting that although antibiotic regulation within the NHS is relatively well developed, the growth of antibiotic use outside the NHS system has become one of the main factors undermining the overall effectiveness of AMR control.

In addition, the environmental domain remains a weak link in the UK's AMR governance framework. The 2024–2029 National Action Plan states that environmental health and AMR surveillance are among the key areas where major breakthroughs are possible but urgent strengthening is required. It notes that the UK's human health data systems are widely recognized internationally, whereas veterinary health data systems, One Health data systems, and data linkage remain relatively underdeveloped. A 2023 report by the environmental authorities likewise pointed out that, although AMR surveillance methods in the clinical and veterinary sectors are relatively mature, methods for environmental AMR surveillance have not yet been fully established, making this area one of the weaker components of AMR surveillance and control. The UK also exhibits clear regional and socioeconomic inequalities in AMR burden. According to ESPAUR 2024–2025, the rate of antimicrobial-resistant bloodstream infections among the most deprived populations was 43.3 cases per 100,000 population, compared with 29.4 cases per 100,000 among the least deprived populations, which is 32.1% lower than that of the former group. These findings indicate that AMR prevention and control in the UK is also affected by regional disparities and inequities between socioeconomically disadvantaged and affluent populations.

### Comparative analysis and implications of AMR governance practices in China and the UK

From a One Health perspective, China and the UK have developed two distinct models of AMR prevention, control, and governance. In responding to the global challenge of AMR containment, the practices of the two countries provide complementary insights, each with its own distinctive strengths. China has established a relatively complete hierarchical governance system, relying on strong administrative capacity to implement a vertically integrated and centrally coordinated model. However, its mechanisms for cross-sectoral collaboration and data sharing are still being further refined. At the same time, China's governance system demonstrates stronger potential for centralized national planning and coordination, which is conducive to rapid, efficient, and unified action during major public health emergencies.

The UK, by contrast, has adopted a model led by specialized institutions, implementing a collaborative approach grounded in professional expertise and information sharing. Its AMR strategy, characterized by successive five-year national action plans, is notable for both its continuity and its international influence. One study examining differences in governance and antimicrobial stewardship (AMS) scores across geographic and economic regions found that the global average score was 51.7 out of 100, whereas the UK scored 83 in AMR governance, ranking just below Norway (85) and the United States (84). This suggests that the UK has already become a global frontrunner in policy leadership for AMR prevention and control, and that its experience warrants wider international adaptation and reference (52).

However, cultural differences also shape governance effectiveness. In both China and the UK, the traditional fields of public health, veterinary medicine, and environmental science have long existed as professional “silos.” Their respective knowledge systems, working languages, and professional cultures differ substantially, creating major barriers to cross-sectoral collaboration. In terms of practical outcomes, both countries continue to face important limitations. In particular, both exhibit weaknesses in environmental AMR prevention and control, including the absence of clearly defined surveillance targets and insufficient policy and financial support. In addition, both countries face problems of regional disparities and inequalities across socioeconomic groups. Future AMR control efforts should therefore build on existing strengths while addressing these weaknesses, in order to improve overall implementation effectiveness.

## Future challenges and prospects

In addressing the global challenge of AMR containment, the UK's whole-chain quantitative management and broad societal participation provide a useful model for the systematic reduction of antimicrobial use. China, by contrast, is one of the world's largest producers of livestock and also one of the most populous countries, with highly diverse farming systems and implementation contexts. Consequently, although AMR prevention and control has been elevated to the level of national strategy, China still faces significant challenges in ensuring that governance measures can be effectively extended to large numbers of geographically dispersed farming entities, implemented at the grassroots level, and adapted to substantial regional variation in enforcement capacity. Meeting these challenges will require more precise policy tools, including risk-based AMR surveillance in small-scale and backyard farming systems, targeted stewardship for high-risk pathogens such as carbapenem-resistant gram-negative bacteria, legally defined data-sharing mechanisms across health, agriculture, and environmental sectors, routine evaluation indicators for NAP implementation, and economic incentives or compensation mechanisms that help farms comply with antimicrobial-use restrictions. The UK's more refined and specialized governance model, meanwhile, relies heavily on social compliance and shared public norms. Given the marked differences in cultural and institutional context, the two models are not directly transferable, but they do offer important opportunities for mutual learning.

At the same time, rapid advances in digitalization, informatization, and artificial intelligence are opening new possibilities for One Health governance. These technologies may help break down data silos, improve decision-making efficiency, and reduce long-standing regional and professional barriers. For example, machine learning can be used to analyze public data and to examine how populations with different cultural backgrounds, educational attainment, and social positions respond to surveillance, early warning, and intervention measures, thereby supporting more targeted and adaptive governance strategies. Nevertheless, the effective application of these technologies will depend on overcoming persistent challenges, particularly those related to data standardization, data quality and reliability, and unequal access to digital and hardware infrastructure.
